# Long-term exposure to ambient air pollution and sequential carcinogenesis in the cardia gastric: a cross-sectional study

**DOI:** 10.1186/s12916-025-04582-1

**Published:** 2025-12-22

**Authors:** Juan Zhu, Huanqing Tao, Ning Kang, Sainan Li, Lili Song, Xue Li, Lingbin Du

**Affiliations:** 1 Department of Cancer Prevention, Zhejiang Cancer Hospital, Hangzhou Institute of Medicine (HIM), Chinese Academy of Sciences, Hangzhou, 310022 Zhejiang China; 2 Department of Epidemiology, School of Public Health, Nanjing Medical University, Nanjing, 211166 Jiangsu China; 3 Institute of Reproductive and Child Health/National Health Commission Key Laboratory of Reproductive Health and Department of Epidemiology and Biostatistics/Ministry of Education Key Laboratory of Epidemiology of Major Diseases (PKU), School of Public Health, Peking University Health Science Centre, Beijing, 100191 China; 4 Institute of Reproductive and Child Health, Peking University/Key Laboratory of Reproductive Health, National Health Commission of the People’s Republic of China, Beijing, 100191 China

**Keywords:** Air pollution, Cardia gastric cancer, Disease progression, Mixture exposure

## Abstract

**Background:**

Cardia gastric cancer (CGC) is a distinct and increasingly prevalent malignancy, yet the role of air pollution in its sequential carcinogenesis remains unclear. The present cross-sectional study aimed to evaluate the associations between long-term exposure to ambient air pollution and the progression of precancerous cardia gastric lesions.

**Methods:**

A total of 99,493 participants were enrolled from the National Opportunistic Screening Program for Upper Gastrointestinal Cancer in Zhejiang Province of China from 2022 to 2023. Long-term exposure to six air pollutants including particulate matter (PM_10_), fine particulate matter (PM_2.5_), sulfur dioxide (SO_2_), nitrogen dioxide (NO_2_), ozone (O_3_), and carbon monoxide (CO) was assessed by using ChinaHighAirPollutants (CHAP) dataset. Cardia lesion progression was classified into five histopathological stages based on endoscopic biopsy. Ordinal logistic regression was used to examine pollutant-specific associations, restricted cubic splines (RCS) were applied to assess the potential exposure–response associations, and weighted quantile sum (WQS) regression was performed to evaluate the mixture effects.

**Results:**

All six pollutants were significantly associated with cardia lesion progression. The adjusted odds ratios (95% confidence intervals, 95% CI) per standard deviation increment in 5-year mean concentration were 1.21 (1.17–1.25) for PM_10_, 1.28 (1.23–1.32) for PM_2.5_, 1.17 (1.13–1.21) for SO_2_, 1.07 (1.03–1.10) for NO_2_, 1.16 (1.12–1.20) for O_3_, and 1.36 (1.30–1.41) for CO. RCS analyses indicated non-linear exposure–response patterns for NO_2_, O_3_, and CO. In mixture models, CO and PM_2.5_ were identified as the predominant contributors, with a 10% increase in combined exposure associated with a 10% (95% CI: 7–13%) higher risk of cardia lesion progression. Effect modification analysis revealed that *Helicobacter pylori*-positive individuals were more susceptible to pollution-related progression.

**Conclusions:**

Long-term exposure to air pollutants is associated with the progression of cardia gastric lesions, highlighting the importance of incorporating air quality improvement into cancer prevention strategies.

**Graphical Abstract:**

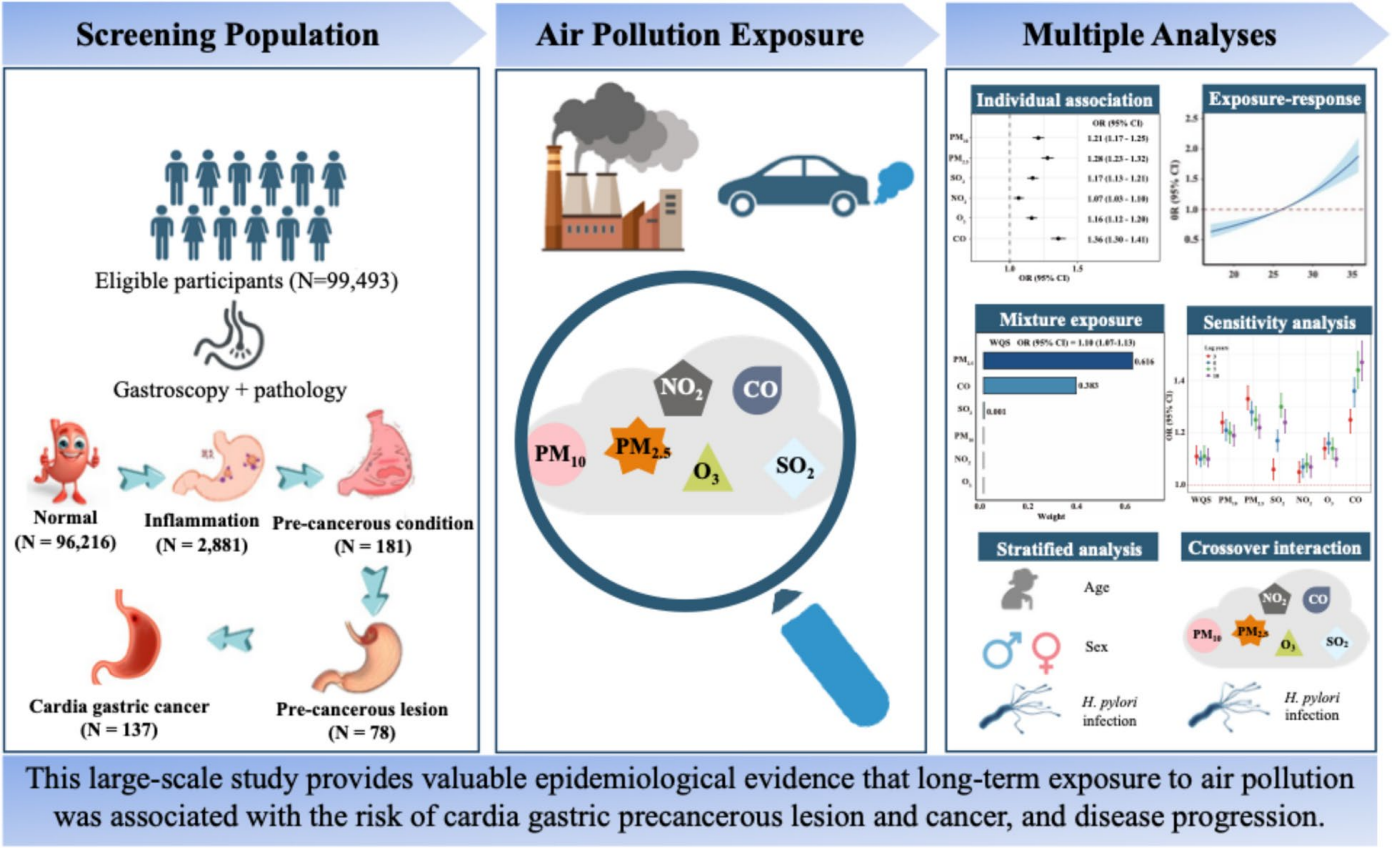

**Supplementary Information:**

The online version contains supplementary material available at 10.1186/s12916-025-04582-1.

## Background

Cardia gastric cancer (CGC) is a distinct subtype of gastric cancer that arises at the esophagogastric junction and exhibits unique epidemiological and pathological features [1–4]. A notable global pattern of gastric cancer has emerged in recent decades. Although the incidence of distal gastric cancer has declined with improved *Helicobacter pylori* (*H. pylori*) control and dietary habits, CGC cases continue to increase, particularly in high-income countries and rapidly industrializing regions such as China [5–7]. The etiological heterogeneity across gastric cancer subtypes provides a key rationale for studying CGC separately. *H. pylori* infection remains the predominant cause of non-cardia gastric cancer [8], whereas its association with CGC is inconsistent [2, 9]. This divergent epidemiological pattern and etiological heterogeneity strongly suggest that, beyond established risk factors, environmental exposures closely linked to industrialization may play a critical role in the pathogenesis of CGC.

The biological plausibility of investigating air pollution as a potential etiological factor for CGC is well supported. Ambient air pollutants, including particulate matter (PM) and gaseous pollutants, have been shown to affect the gastrointestinal system through multiple mechanisms. The International Agency for Research on Cancer (IARC) has classified PM as a group 1 carcinogen, with carcinogenic effects not limited to the respiratory tract [10–13]. Inhaled fine particles (PM_2.5_) can penetrate the alveolar-capillary barrier and enter systemic circulation, reaching distal organs such as the stomach [14–17]. This systemic exposure may activate a “pollution-gut axis,” whereby pollutants induce oxidative stress, chronic inflammation, and DNA damage in the gastric mucosa, ultimately disrupting tissue homeostasis and initiating carcinogenesis [18, 19]. Additionally, pollutants may enter the gastrointestinal tract via ingestion or mucociliary clearance. Owing to its unique anatomical position, the gastric cardia may be simultaneously affected by both pathways, rendering it particularly susceptible to the toxic effects of air pollution.

Although existing epidemiological studies have provided preliminary evidence linking air pollution to overall gastric cancer risk [20–22], several critical gaps remain. First, most previous studies have treated gastric cancer as a homogeneous disease without distinguishing between anatomical subtypes such as cardia and non-cardia cancer [23–25]. Given CGC’s distinct etiologic profile, such an undifferentiated approach may obscure or dilute its specific associations with air pollution. Second, prior research has largely focused on particulate matter, with limited investigation of gaseous pollutants such as sulfur dioxide (SO_2_), nitrogen dioxide (NO_2_), ozone (O_3_), and carbon monoxide (CO), or the combined effects of these pollutants that typically coexist as complex mixtures in real-world settings. Finally, most studies have examined cancer as a binary endpoint, overlooking the dynamic, multistage process of gastric carcinogenesis—from normal mucosa through chronic inflammation, precancerous conditions, and precancerous lesions to malignancy. Understanding whether air pollution contributes to earlier or intermediate stages of this continuum is crucial for elucidating carcinogenic mechanisms and identifying opportunities for early intervention.

Based on these knowledge gaps and biological plausibility, we propose the following hypothesis: long-term exposure to ambient air pollution is independently and dose-dependently associated with the progression of cardia lesions, and this association may be synergistically modified by *H. pylori* infection. Accordingly, this large-scale cross-sectional study aims to (1) systematically evaluate the associations between long-term exposure to six major air pollutants (PM_10_, PM_2.5_, SO_2_, NO_2_, O_3_, and CO) and the full spectrum of cardia lesion progression—from normal mucosa, inflammation, precancerous conditions, and precancerous lesions to cancer; (2) assess the combined effects of multi-pollutant exposure; and (3) elucidate the potential modifying role of *H. pylori* infection in the relationship between air pollution and cardia lesion progression.

## Methods

### Study population

In our cross-sectional study, participants were enrolled from the Opportunistic Screening Program for Upper Gastrointestinal Cancer in Zhejiang Province from 2022 to 2023, which included 98 hospitals across 11 cities. Eligible participants were adults aged 40–74 years who underwent gastroscopy for opportunistic screening upon hospital admission. Endoscopic anomalies were histologically confirmed through biopsy examination. A total of 122,732 individuals were initially enrolled. We excluded those with incomplete endoscopy (*n* = 29,861) or missing baseline covariates (*n* = 430), resulting in a final analytical sample of 99,493 participants. Ethical approval was obtained from the Zhejiang Cancer Hospital Institutional Review Board (IRB-2023–890), and all participants provided written informed consent.

### Exposure assessment

Long-term exposure to six air pollutants, including PM_10_, PM_2.5_, SO_2_, NO_2_, O_3_, and CO, was obtained from the ChinaHighAirPollutants (CHAP) dataset (available at https://weijing-rs.github.io/product.html). CHAP dataset integrates satellite remote sensing, ground monitoring, atmospheric reanalysis, and chemical transport modeling to provide high-resolution pollutant concentrations [26–28]. In the present study, each participant was assigned monthly concentrations based on the geocoded residential address. For each pollutant, we calculated the 5-year (60-month) average concentration preceding the month of survey for each participant, representing the lagged long-term exposure prior to baseline. Alternative exposure windows (3-, 7-, and 10-year averages) were applied in sensitivity analyses.

### Outcome definitions

Upper endoscopy with histopathological confirmation was used to determine disease status. Based on the natural history of cardia disease progression, participants were classified into five mutually exclusive categories: “normal” indicated histologically normal cardia mucosa; “inflammation or polyp” referred to non-atrophic carditis or benign hyperplastic polyp; “pre-cancerous condition” was defined as the presence of atrophic carditis or intestinal metaplasia; “pre-cancerous lesion” included low-grade intraepithelial neoplasia (LGIN) and high-grade intraepithelial neoplasia (HGIN); and “cardia gastric cancer” was confirmed according to the International Classification of Diseases, 10th Revision (ICD-10) code C16.0. Cardia lesion progression was defined as a histological transition to a higher grade, following the biological continuum. Participants with stable histological categories were considered non-progressors. In cases where multiple lesions were detected, classification was based on the most advanced histological finding.

### Covariates

Baseline covariates were collected through standardized questionnaires, including age (< 50, 50–59, 60–74 years), sex (female, male), body mass index (BMI, < 18.5, 18.5–23.9, 24.0–27.9, and ≥ 28.0 kg/m^2^), education level (elementary school or below, junior or senior high school, college and above), smoking status (never, ever), alcohol drinking status (never, ever), hot food and tea preference (no, yes), frequency of consumption of preserved foods (≥ 4 days/week, < 4 days/week), frequency of consumption of fresh vegetables (≥ 4 days/week, < 4 days/week), and *H. pylori* infection status (negative, positive).

### Statistical analyses

Baseline characteristics were summarized by gastric cardia lesion status. Continuous variables were presented as means with standard deviation (SD), while categorical variables were described as numbers with percentages. Differences across lesion categories were compared using analysis of variance (ANOVA) for continuous variables and chi-square tests for categorical variables. Variables showing statistically significant differences (*P* < 0.05) were adjusted for in multivariable models. Spearman correlation coefficients were used to examine intercorrelations among the six pollutants.

We adopted a progressive modeling strategy to evaluate the association between air pollution and cardia lesion progression. First, ordinal logistic regression models were fitted to estimate odds ratios (ORs) and 95% confidence intervals (CIs) for lesion severity per one-SD increase in pollutant concentration. The proportional odds assumption was examined using the Brant test. Second, considering that non-linear exposure–response relationships have been widely reported in prior air pollution studies, we applied restricted cubic spline (RCS) models to assess potential non-linear associations. Non-linearity was evaluated using likelihood ratio tests. Third, given the high intercorrelations among pollutants, weighted quantile sum (WQS) regression was performed to examine the mixture effects [29]. The model specified four quantiles and 500 bootstrap iterations to obtain 95% CIs and to estimate pollutant-specific weights representing their relative contributions to the overall mixture.

To enhance the robustness of our findings, we performed several sensitivity analyses. First, pollutant concentrations were categorized into quartiles (Q1–Q4) to verify consistency of exposure–response patterns. Second, because the Brant test is highly sensitive to large sample sizes, we additionally modeled lesion severity as a continuous outcome, assuming equal or proportional spacing among stages, and fitted linear regression models to assess consistency in direction and magnitude. Third, we redefined the exposure window as 3-, 7-, and 10-year averages before baseline to test temporal robustness. Additionally, participants enrolled in 2022–2023 were excluded to assess temporal stability of the results.

Subgroup analyses were further conducted by age, sex, and *H. pylori* infection status to explore effect modification. Additive interactions between *H. pylori* infection and high pollutant exposure were quantified using the relative excess risk due to interaction (RERI), attributable proportion (AP), and synergy index (SI), with 95% CIs derived by the delta method. Values of RERI = 0, AP = 0, and SI = 1 indicate no interaction, whereas RERI > 0, AP > 0, and SI > 1 denote positive (synergistic) interaction.

All statistical analyses were performed using R version 4.4.2 (R Foundation for Statistical Computing, Vienna, Austria), and two-tailed *P* values < 0.05 were considered statistically significant.

## Results

### Study populations

Table 1 presents the baseline characteristics of the study participants. A total of 99,493 participants were included in this study, with a mean age of 57.28 years (SD = 8.62 years). Among those, 2881 cases of inflammation and polyps (2.89%), 181 pre-cancerous conditions (0.18%), 78 pre-cancerous lesions (0.08%), and 137 cancer cases (0.14%) were diagnosed by gastroscopy and pathology. Significant differences were observed in the distribution of age (*P* < 0.001), sex (*P* < 0.001), and *H. pylori* infection (*P* = 0.041) across the different cardia disease states. Patients with cardia lesions were older, male, and more likely to have positive *H. pylori* infection, while other lifestyle and demographic factors showed no significant difference across disease stages.

**Table 1:** Baseline characteristics of participants by status of cardia diseases

Characteristics	Overall (*N* = 99,493)	Normal (*N* = 96,216)	Inflammation or polyp (*N* = 2881)	Pre-cancerous condition (*N* = 181)	Pre-cancerous lesion (*N* = 78)	Cardia gastric cancer (*N* = 137)
Age, mean (SD)	57.28 (8.62)	57.24 (8.62)	58.09 (8.56)	60.82 (8.21)	61.94 (8.15)	65.06 (6.46)
Age group, *n* (%)						
< 50	20,433 (20.54)	19,897 (20.68)	513 (17.81)	17 (9.39)	4 (5.13)	2 (1.46)
50–59	38,890 (39.09)	37,704 (39.19)	1074 (37.28)	62 (34.25)	26 (33.33)	24 (17.52)
60–74	40,170 (40.37)	38,615 (40.13)	1294 (44.92)	102 (56.35)	48 (61.54)	111 (81.02)
Sex, *n* (%)						
Female	52,420 (52.69)	50,999 (53.00)	1311 (45.51)	63 (34.81)	17 (21.79)	30 (21.90)
Male	47,073 (47.31)	45,217 (47.00)	1570 (54.49)	118 (65.19)	61 (78.21)	107 (78.10)
BMI, kg/m^2^, *n* (%)						
< 18.5	2699 (2.71)	2617 (2.72)	70 (2.43)	6 (3.31%)	3 (3.85%)	3 (2.19%)
18.5–23.9	59,870 (60.18)	57,860 (60.14)	1766 (61.30%)	98 (54.14%)	54 (69.23%)	92 (67.15%)
24.0–27.9	32,322 (32.49)	31,278 (32.51)	931 (32.32%)	63 (34.81%)	16 (20.51%)	34 (24.82%)
≥ 28.0	4602 (4.63)	4461 (4.64)	114 (3.96%)	14 (7.73%)	5 (6.41%)	8 (5.84%)
Education level, *n* (%)						
Elementary school or below	44,770 (45.00)	43,357 (45.06)	1234 (42.83)	81 (44.75)	38 (48.72)	60 (43.80)
Junior or senior high school	44,597 (44.82)	43,073 (44.77)	1342 (46.58)	82 (45.30)	35 (44.87)	65 (47.45)
College and above	10,126 (10.18)	9786 (10.17)	305 (10.59)	18 (9.94)	5 (6.41)	12 (8.76)
Smoking status, *n* (%)						
Never	82,710 (83.13)	79,974 (83.12)	2401 (83.34)	154 (85.08)	63 (80.77)	118 (86.13)
Ever	16,783 (16.87)	16,242 (16.88)	480 (16.66)	27 (14.92)	15 (19.23)	19 (13.87)
Drinking status, *n* (%)						
Never	82,855 (83.28)	80,117 (83.27)	2396 (83.17)	155 (85.64)	65 (83.33)	122 (89.05)
Ever	16,638 (16.72)	16,099 (16.73)	485 (16.83)	26 (14.36)	13 (16.67)	15 (10.95)
Hot food and tea preference, *n* (%)						
No	79,144 (79.55)	76,562 (79.57)	2260 (78.44)	155 (85.64)	58 (74.36)	109 (79.56)
Yes	20,349 (20.45)	19,654 (20.43)	621 (21.56)	26 (14.36)	20 (25.64)	28 (20.44)
Frequency of consumption of preserved foods, *n* (%)						
≥ 4 days/week	17,753 (17.84)	17,225 (17.90)	468 (16.24)	24 (13.26)	11 (14.10)	25 (18.25)
< 4 days/week	81,740 (82.16)	78,991 (82.10)	2413 (83.76)	157 (86.74)	67 (85.90)	112 (81.75)
Frequency of consumption of fresh vegetables, *n* (%)						
≥ 4 days/week	44,057 (44.28)	42,623 (44.30)	1257 (43.63)	81 (44.75)	33 (42.31)	63 (45.99)
< 4 days/week	55,436 (55.72)	53,593 (55.70)	1624 (56.37)	100 (55.25)	45 (57.69)	74 (54.01)
*H. pylori* infection, *n* (%)						
Negative	69,689 (70.04)	67,405 (70.06)	2007 (69.66)	139 (76.80)	55 (70.51)	83 (60.58)
Positive	29,804 (29.96)	28,811 (29.94)	874 (30.34)	42 (23.20)	23 (29.49)	54 (39.42)

Pre-cancerous condition: atrophic gastritis and intestinal metaplasia

Pre-cancerous lesion: low-grade intraepithelial neoplasia (LGIN) and high-grade intraepithelial neoplasia (HGIN)

*SD* standard deviation

**Fig. 1:**
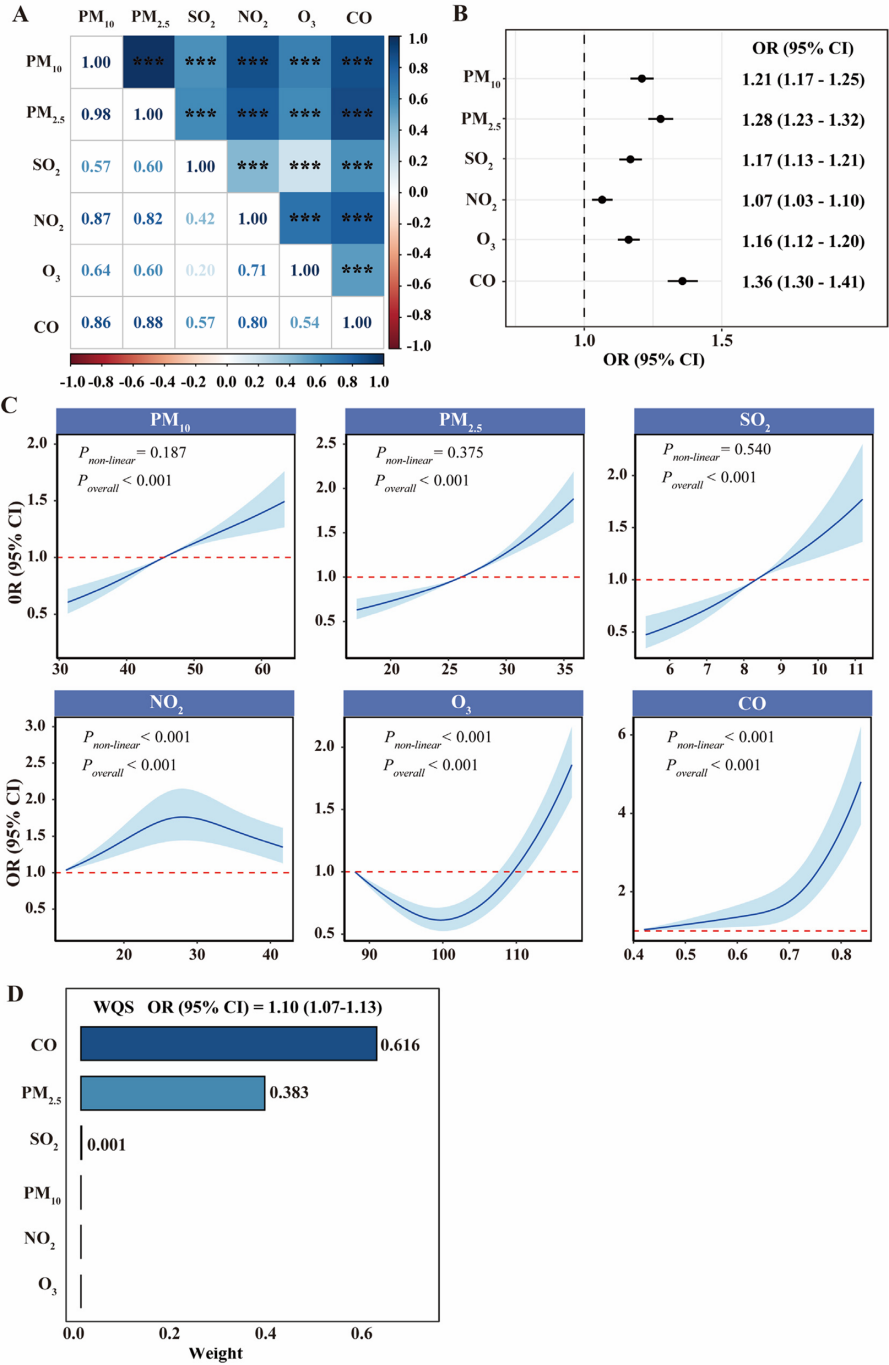
Association of individual and mixture air pollutants exposure with cardia gastric cancer. **A** Correlation between 5-year averaged concentrations of air pollutants among participants. **B** Associations between 5-year averaged concentrations of air pollutants and cardia disease progression using ordinal logistic regression, estimated in 1 standard deviation (SD) increments. **C** Exposure–response associations of 5-year average concentration of air pollutants and cardia disease progression. **D** The overall effect of air pollutant mixture (estimates and 95% CI) on cardia disease progression. Weighted quantile sum (WQS) analysis estimated weights of six air pollutants in the associations with cardia disease progression

The 5-year average concentrations of six air pollutants are presented in Additional file 1: Table S1. The mean (SD) exposure concentrations of PM_10_, PM_2.5_, SO_2_, NO_2_, O_3_, and CO were 46.21 ± 6.90 μg/m^3^, 26.39 ± 4.14 μg/m^3^, 8.32 ± 0.70 μg/m^3^, 26.74 ± 6.20 μg/m^3^, 100.52 ± 6.10 μg/m^3^, and 0.69 ± 0.07 mg/m^3^, respectively. The means for PM_2.5_ and PM_10_ exceeded the guidelines established by the World Health Organization (Air Quality Guidelines 2021, PM_2.5_: 5 μg/m^3^, PM_10_: 10 μg/m^3^) [30, 31]. The average concentrations of air pollutants varied significantly across different types of cardia lesions. Figure 1A illustrates correlations of air pollutants among participants, which exhibited strong positive correlations among PM_10_, PM_2.5_, NO_2_, and CO, with Spearman correlation coefficients ranging from 0.82 to 0.98. In contrast, the correlations between SO_2_ and the other five air pollutants were relatively weak, with coefficients ranging from 0.20 to 0.60.

### Associations of air pollutants with cardia lesions

Figure 1B presents the associations between air pollution exposure (continuous variables) and cardia lesion progression by multivariable ordinal logistic regression. After adjustment for covariates (age, sex, and *H. pylori* infection), models revealed that six air pollutants were positively associated with cardia lesion progression. The ORs (95% CI) per SD increase in 5-year average concentration were 1.21 (1.17–1.25) for PM_10_, 1.28 (1.23–1.32) for PM_2.5_, 1.17 (1.13–1.21) for SO_2_, 1.07 (1.03–1.10) for NO_2_, 1.16 (1.12–1.20) for O_3_, and 1.36 (1.30–1.41) for CO, respectively.

Figure 1C depicts the exposure–response associations for all pollutants related to cardia disease progression. Exposure–response analyses of PM_10_, PM_2.5_, and SO_2_ showed a monotonic risk increase, with no evident thresholds (*P*_*non-linear*_ > 0.05). Conversely, NO_2_, O_3_, and CO exhibited clear non-linear associations with cardia lesion progression (*P*_*non-linear*_ < 0.001). A 10% increase in the concentrations of all six air pollutants was associated with a 10% increase in the risk of cardia disease progression (95% CI: 7–13%) (Fig. 1D). The WQS regression model attributed the largest regression weight to CO (0.616), followed by PM_2.5_ (0.383).

### Subgroup and interaction analysis

**Fig. 2:**
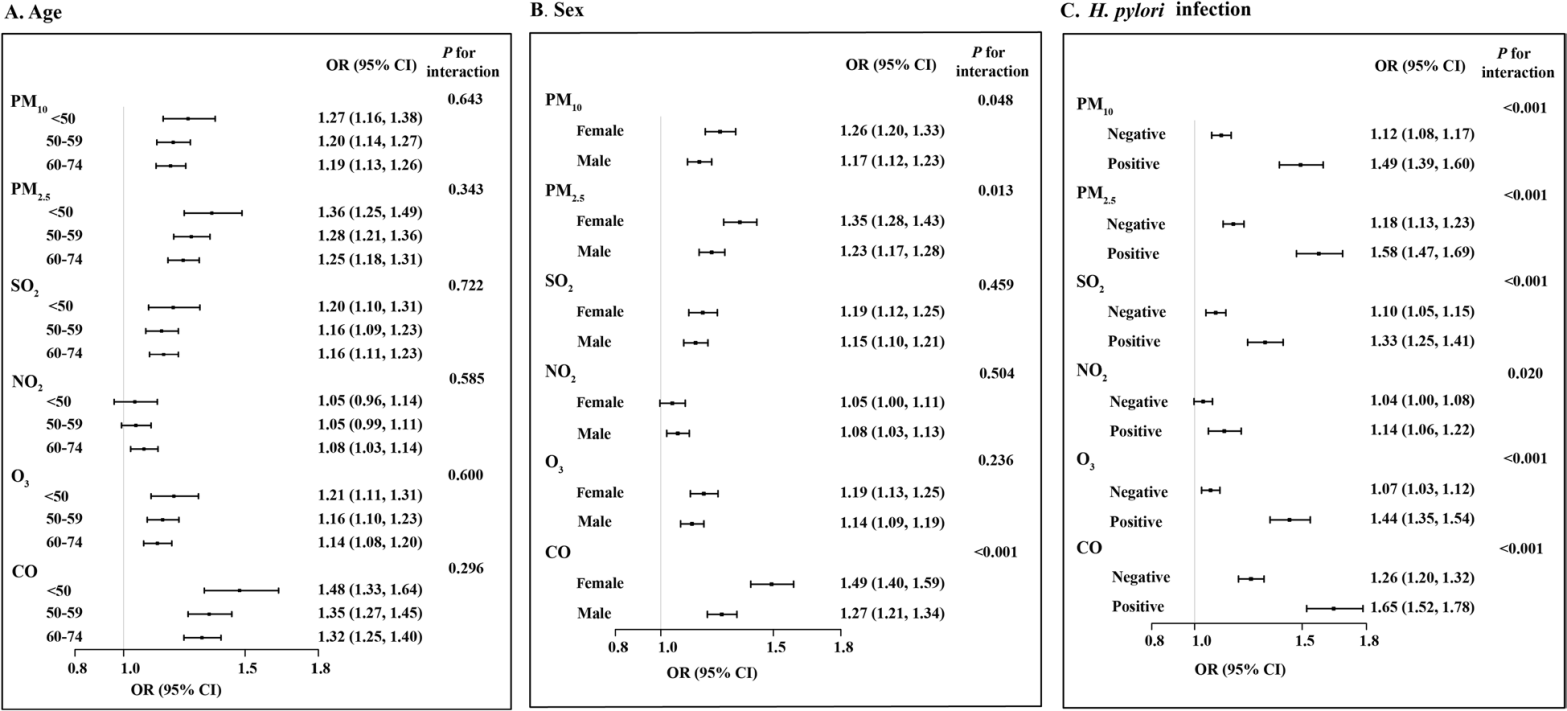
Association of 5-year averaged concentrations of air pollutants exposure with cardia disease progression, stratified by **A** age, **B** sex, and **C** *H. pylori* infection. Model adjusted for age, sex, and *H. pylori* infection except for the stratified variable

Stratified analyses revealed that air pollution’s association with gastric cardia lesion progression persisted across age groups, sexes, and *H. pylori* infection status (Fig. 2). Although odds ratios for individual pollutants (PM_10_, PM_2.5_, SO_2_, O_3_, CO) varied slightly with age, all age strata showed an elevated risk of cardia lesions. Take PM_10_ for example, the association was strongest in the youngest group (< 50 years, OR: 1.27, 95% CI: 1.16–1.38), with slightly attenuated but still significant associations in older groups (50–59 years, OR: 1.20, 95% CI: 1.14–1.27; 60–74 years, OR: 1.19, 95% CI: 1.13–1.26). Interaction analyses found no significant modification of pollutant effects by age. In contrast, women exhibited greater susceptibility to PM_10_, PM_2.5_, and CO (*P* for interaction < 0.05). Specifically, for CO exposure, the associated risk was significantly higher in females (OR: 1.49, 95% CI: 1.40–1.59) than in males (OR: 1.27, 95% CI: 1.21–1.34). *H. pylori*-positive status significantly amplified the impact of all six pollutants (all *P* for interaction < 0.05).

Additive interaction analyses (Table 2) dichotomized each pollutant at the control median. Positive additive interactions (RERI > 0 and AP > 0) indicated synergistic interactions between *H. pylori* infection and high exposure to PM_10_, PM_2.5_, SO_2_, O_3_, and CO. In the absence of pollution (R01), *H. pylori* infection alone showed ORs < 1 for these pollutants, suggesting a potential inverse association between *H. pylori* infection and gastric cardia cancer risk. Pollution alone (R10) yielded ORs > 1 (except for O_3_), confirming air pollution as an independent risk factor. In joint exposure (R11), although some ORs did not reach significance, positive RERI and AP values demonstrated that combined *H. pylori* infection and high pollutant exposure exerted a greater than additive risk, supporting a synergistic model.

**Table 2:** Analysis of the interaction between *H. pylori* infection and air pollutants in relation to the risk of cardia lesion progression

	Variable		OR (95%CI)
	High PM_10_	*H. pylori* infection	
R00	−	−	1.00 (reference)
R01	−	+	0.77 (0.60–0.99)
R10	+	−	1.25 (1.15–1.36)
R11	+	+	1.14 (0.97–1.35)
RERI			0.12 (0.02, 0.22)
AP			0.10 (0.01, 0.20)
SI			6.24 (0.00, 1.93*10^5^)
	High PM_2.5_	*H. pylori* infection	
R00	−	−	1.00 (reference)
R01	−	+	0.64 (0.50, 0.82)
R10	+	−	1.22 (1.12, 1.33)
R11	+	+	1.06 (0.89, 1.25)
RERI			0.20 (0.12, 0.27)
AP			0.19 (0.09, 0.28)
SI			NA
	High SO_2_	*H. pylori* infection	
R00	−	−	1.00 (reference)
R01	−	+	0.57 (0.44, 0.73)
R10	+	−	1.15 (1.05–1.25)
R11	+	+	0.94 (0.79–1.11)
RERI			0.22 (0.15–0.29)
AP			0.24 (0.13–0.34)
SI			NA
	High NO_2_	*H. pylori* infection	
R00	−	−	1.00 (reference)
R01	−	+	0.94 (0.73–1.20)
R10	+	−	1.21 (1.11–1.31)
R11	+	+	1.18 (1.00–1.39)
RERI			0.03 (− 0.09, 0.16)
AP			0.03 (− 0.08, 0.14)
SI			1.24 (0.42, 3.67)
	High O_3_	*H. pylori* infection	
R00	−	−	1.00 (reference)
R01	−	+	0.66 (0.52, 0.84)
R10	+	−	0.93 (0.86, 1.01)
R11	+	+	0.81 (0.69, 0.95)
RERI			0.22 (0.12, 0.31)
AP			0.27 (0.12, 0.42)
SI			NA
	High CO	*H. pylori* infection	
R00	−	−	1.00 (reference)
R01	−	+	0.69 (0.54, 0.88)
R10	+	−	1.21 (1.12, 1.32)
R11	+	+	1.08 (0.91, 1.27)
RERI			0.17 (0.09, 0.26)
AP			0.16 (0.06, 0.26)
SI			NA

Each air pollutant was dichotomized into high and low exposure groups based on the median concentration in the control group

Crossover analysis was employed to examine potential additive interactions between serum air pollutant levels and *H. pylori* infection in relation to the risk of cardia cancer and precancerous lesions

*RERI* Relative excess risk due to interaction, *AP* Attributable proportion to interaction, *SI* Synergy index

### Sensitivity analysis

Sensitivity analysis results of cardia lesion progression corresponding to the air pollutant quartiles are illustrated in Additional file 1: Table S2. Consistently, compared with the first quartile, the adjusted OR (95% CI) of cardia lesion progression in the highest quartile were increased for all six pollutants: 1.69 (1.53–1.87) for PM_10_, 1.88 (1.70–2.07) for PM_2.5_, 1.44 (1.30–1.60) for SO_2_, 1.12 (1.01–1.24) for NO_2_, 1.30 (1.18–1.42) for O_3_, and 1.82 (1.64–2.00) for CO, respectively.

**Fig. 3:**
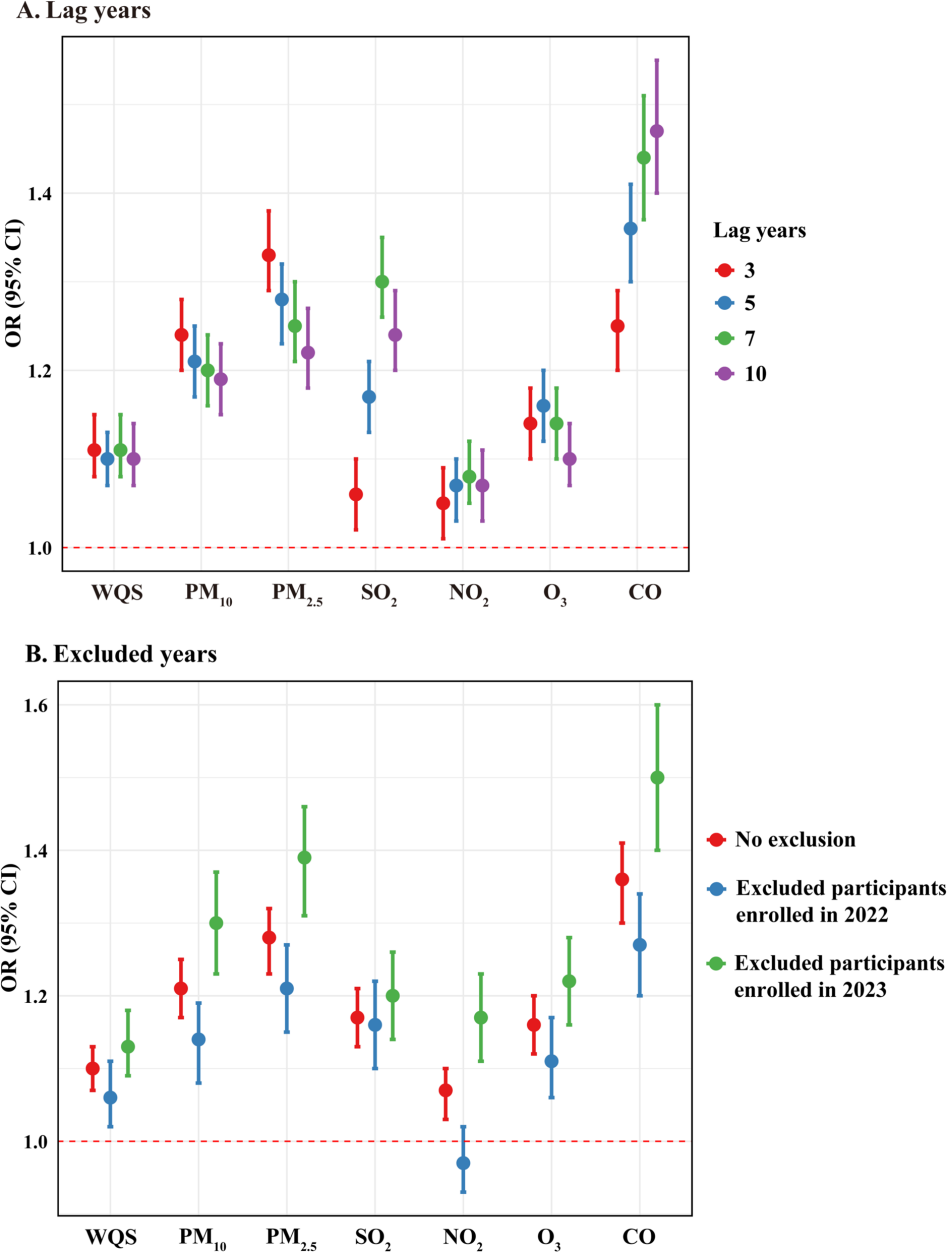
Sensitivity analysis of the association between air pollutant exposure and cardia disease progression. **A** In different exposure windows. **B** After excluding different years of enrollment

As shown in Fig. 3, neither different exposure windows nor the exclusion of participants from different enrollment years changed the associations of air pollution exposure and cardia lesion progression, further reinforcing the consistency and reliability of the findings (Additional file 1: Tables S3 and S4). Besides, assuming equal or proportional spacing among stages, fitted linear regression consistently confirmed that air pollutants were positively associated with cardia lesion progression, except for NO_2_ (Additional file 1: Table S5).

## Discussion

This large-scale study provides robust evidence that long-term exposure to ambient air pollution, including PM_10_, PM_2.5_, SO_2_, NO_2_, O_3_, and CO, is independently and synergistically associated with a graded increase in the progression of cardia lesions along the inflammation-precancerous-carcinoma sequence. Adjusted ORs per SD increase ranged from 1.07 (NO_2_) to 1.36 (CO). Combined pollutant exposure was linked to a 10% higher risk of lesion progression, with CO and PM_2.5_ contributing most to this mixture effect. Females and those with *H. pylori* infection demonstrated heightened susceptibility to the effects of air pollution. Additionally, we observed significant additive interactions between high exposure levels of PM_10_, PM_2.5_, SO_2_, O_3_, and CO and *H. pylori* infection in relation to the risk of cardia lesion progression. These findings provide the first histologically anchored and epidemiological evidence that air pollution acts as a multistage carcinogenic promoter in cardia gastric tumorigenesis, offering critical insights for environmentally informed cancer control policies.

While large European cohorts and Chinese studies have linked air pollution to overall gastric cancer risk [21, 32], the ELAPSE study and a recent meta-analysis failed to replicate these associations [33, 34]. These inconsistencies underscore the complexity of disentangling environmental influences on gastric carcinogenesis and highlight the limitations of studies that treat gastric cancer as a homogeneous entity. Notably, most prior investigations did not distinguish between gastric cancer subtypes, thereby potentially masking subtype-specific associations. CGC, which arises at the proximal stomach near the esophagogastric junction, differs markedly from non-cardia gastric cancer in terms of etiology, histopathology, and risk factors. For instance, *H. pylori* is a well-established driver of non-cardia cancer but plays a less consistent role in CGC [9, 35], suggesting that CGC’s environmental risk factors may operate through distinct mechanisms. Thus, lumping CGC together with distal gastric cancers in epidemiologic analyses may have diluted or obscured pollutant-specific associations relevant to the cardia.

This study innovatively introduces a histopathological progression model, systematically elucidating the multistage carcinogenic role of ambient air pollution in cardia lesions. Departing from the conventional binary definition of cancer, our study incorporated a biologically and clinically meaningful continuum of gastric cardia carcinogenesis, spanning normal mucosa, inflammation, precancerous changes, and malignancy. Results revealed 7–36% increased odds of lesion progression per SD pollutant increment, with CO demonstrating gradient associations (OR = 1.36). These findings not only demonstrate a clear dose–response pattern but also validate the biological plausibility of cumulative damage caused by chronic pollutant exposure. By anchoring pollutant effects to distinct histological transitions, our study goes beyond endpoint-based (cancer-diagnosis) risk estimations and provides compelling evidence of progressive, stage-specific environmental injury in the cardia. This integrated framework offers several novel contributions. Epidemiologically, it enhances causal inference by aligning exposure associations with known cancer progression biology. Clinically, it provides a conceptual foundation for early risk stratification and targeted intervention, particularly for individuals in reversible or intermediate stages. From a policy perspective, it supports the refinement of air quality guidelines by demonstrating that even sub-threshold exposures can initiate early carcinogenic changes, emphasizing the need for stricter, health-oriented environmental standards.

Potential mechanisms linking air pollution to gastric cardia cancer are unclear, but several studies have provided crucial evidence. Fine particulate matter and gaseous pollutants can reach the gastric cardia through multiple pathways: direct ingestion from the oropharynx, mucociliary transport into the gastrointestinal tract, or indirect translocation from the alveolar-capillary barrier into systemic circulation. Once deposited, these pollutants generate reactive oxygen species (ROS), inflicting direct DNA damage and inducing mutations in key tumor suppressor genes such as TP53 and CDKN2A, which are frequently implicated in cardia carcinogenesis [36]. Chronic exposure to air pollution also triggers systemic and mucosal inflammation, elevating cytokines and disrupting the epithelial barrier. The cardia, with its dense submucosal capillary network, may be particularly susceptible to these circulating inflammatory mediators [37, 38]. Furthermore, inhaled particles can alter the gut microbiome, compromising mucosal immunity and promoting pro-tumorigenic environments [39].

Particulate matter (PM_2.5_ and PM_10_), classified as group 1 carcinogens by the International Agency for Research on Cancer (IARC), exerts its toxicity not only through intrinsic properties but also by serving as vectors for adsorbed toxicants such as polycyclic aromatic hydrocarbons (PAHs) and heavy metals. These compounds penetrate cellular compartments—cytoplasm, mitochondria, and nuclei—activating proliferative signaling, suppressing apoptosis, and exacerbating oxidative stress [40–42]. NO_2_ and O_3_, both traffic- and combustion-related pollutants, have been associated with increased cancer mortality and may induce pro-inflammatory signaling and DNA strand breaks in epithelial cells [43–45]. SO_2_, mainly from coal and industrial emissions, generates acidic metabolites that damage gastric mucosa and induce chronic inflammation. It also activates oxidative stress-responsive transcription factors (e.g., AP-1, NF-κB), upregulating IL-8 and MMPs to facilitate epithelial-mesenchymal transition and tumor invasion [46]. Short-term spikes in ambient SO_2_ have also been linked to increased mortality risk [47]. CO binds to hemoglobin, exacerbating hypoxia and disrupting mitochondrial function, which impairs ATP synthesis and increases ROS levels, thereby compromising DNA repair and promoting malignant transformation [48, 49]. Collectively, these pollutants drive multistage carcinogenesis in the gastric cardia through interconnected mechanisms, including oxidative damage, chronic inflammation, immune dysregulation, epigenetic changes, and metabolic alterations. Our findings not only reinforce the plausibility of these mechanistic pathways but also underscore the urgency of integrated public health strategies to reduce environmental exposures and mitigate cancer risk at the population level.

Identifying susceptible subpopulations is essential for tailoring public health interventions to reduce disparities in cardia gastric cancer burden. Our findings highlight greater pollutant-related vulnerability in females, consistent with prior evidence suggesting that estrogen modulates systemic inflammation and xenobiotic metabolism by influencing phase I/II detoxification enzymes (e.g., *CYP1A1*, *GSTM1*) and antioxidant defenses [45, 50–52]. Sex-specific immune responses, such as heightened Th17-driven inflammation and estrogen-mediated cross-talk with inflammatory pathways (e.g., NF-κB, JAK/STAT), may further amplify pollutant-induced effects [53, 54].

*H. pylori* is a well-established etiologic agent for non-cardia gastric cancer, but its role in cardia cancer is highly heterogeneous and remains debated [2]. Several East Asian studies report a modest positive association between *H. pylori* infection and CGC, whereas most investigations in Europe, North America, and Australia observe null or even inverse relationships [9, 35]. Our study revealed a synergistic interaction between air pollution and *H. pylori* infection, supporting a “dual-hit” model whereby environmental and microbial insults converge to accelerate cardia carcinogenesis [55]. Mechanistically, *H. pylori* creates a pro-inflammatory niche marked by IL-8 and TNF-α upregulation, priming the mucosa for ROS overproduction upon pollutant exposure [56]. Particulate matter disrupts epithelial tight junctions, promoting *H. pylori* adhesion and deeper mucosal infiltration. This process initiates a cycle of oxidative damage and enhances microbial virulence. These findings underscore the potential for targeted strategies, such as prioritizing *H. pylori* eradication in high-pollution areas, to mitigate the compounded risk of cardia lesions. Future studies are needed to elucidate the mechanistic pathways involved in this interaction.

Our findings have important policy implications. First, reducing air pollution offers an effective, equitable strategy to alleviate the burden of CGC and promote regional health equity. In China, where the gastric cancer burden remains prevalent, current efforts emphasize healthy lifestyles and expanded cancer screening programs [57]. However, these interventions often rely heavily on individual participation and may inadvertently exacerbate health disparities in socioeconomically disadvantaged areas with limited access. In contrast, improving air quality is a population-level intervention that can equitably reduce exposure and disease risk, thus supporting health equity [58]. Second, sustained efforts in air pollution control are critical. Although China has made significant progress in reducing emissions, population-weighted PM_2.5_ still exceeds WHO air quality guidelines. Given no safe threshold for PM_2.5_ and PM_10_ exposure, stricter air quality regulations are necessary to mitigate their population-level impact. Challenges persist, including coal dependency, uneven regional coordination, and the need to balance economic growth with emission reduction. Future strategies should prioritize energy-sector transformation, expansion of carbon markets, enhanced monitoring infrastructure, and technological innovation to ensure carbon peaking targets by 2030 and carbon neutrality by 2060 [59]. Third, air quality policies should be explicitly health-driven. Evidence indicates that health-centered strategies can substantially reduce population-weighted toxic exposures up to 5.4-fold compared to conventional approaches [60]. Therefore, we advocate for cost-effective, health-oriented air pollution policies that maximize public health benefits.

To our knowledge, this is the first large-scale study to comprehensively evaluate both individual and combined effects of particulate (PM_2.5_, PM_10_) and gaseous pollutants (SO_2_, NO_2_, O_3_, CO) across the entire spectrum of gastric cardia carcinogenesis (spanning inflammation, polyps, precancerous conditions, lesions, and carcinoma), using endoscopy-confirmed outcomes in nearly 100,000 adults in China. By integrating precise anatomical localization at the gastroesophageal junction with a dynamic, stage-based pathological carcinogenesis model, this study fills a critical gap in evidence linking environmental exposures to anatomically distinct cancer subtypes. By employing multiple modeling approaches, including ordinal logistic regression, exposure–response spline analyses, and WQS mixture modeling, we were able to reliably characterize the real-world effects of pollutants. Sensitivity analyses across varying exposure durations and case exclusions confirmed the robustness of findings. These results underscore air pollution as a modifiable environmental risk factor in cardia carcinogenesis, with direct implications for cancer prevention and public health policy.

Several limitations should be mentioned. First, exposure misclassification remains a concern, as exposure assessment was based on geocoded residential addresses, which may not fully reflect individual-level exposures due to behavioral variability (e.g., residential relocation). While this approach may not fully capture individual-level variability in exposure, it remains a reliable method for large-scale epidemiological studies. We mitigated misclassification by restricting analyses to permanent residents and conducting sensitivity tests across different exposure windows to account for tumor latency. Second, given the significant correlations among certain pollutants, isolating the independent effects of each pollutant is challenging. Future studies should consider more precise exposure assessment methods, such as personal exposure monitoring and advanced statistical techniques, to further elucidate the independent effects of individual pollutants. Third, as an observational study, our design cannot prove causality, and further longitudinal cohort studies and mechanistic investigations are required to confirm these findings and explore potential causal pathways. While causality remains unproven, our study strengthens the epidemiological evidence linking air pollution to gastric cancer, particularly at the cardia, and provides a foundation for future research. Fourth, given the voluntary nature of the screening program, potential selection bias cannot be ruled out. Such differences may lead to an underestimation of the true association between air pollution and cardia disease progression. Fifth, the Brant test is known to be overly sensitive in large datasets and often detects statistically significant but substantively negligible deviations [61, 62]. Considering that disease progression represents a continuous pathological process and that multiple sensitivity analyses yielded consistent results, we retained the ordinal logistic regression model as the primary analytical framework for its interpretability and comparability. Another limitation of this study is the small number of precancerous or cancer cases. Future studies with longer follow-up periods are needed to accumulate more cases and better assess the impact of long-term air pollution exposure on disease progression. Finally, our cohort was predominantly drawn from Zhejiang Province, which may limit generalizability to regions with different pollution profiles. Nonetheless, considering that evidence is rare, our results could still provide important insights.

## Conclusions

This study reveals that long-term exposure to ambient particulate and gaseous pollutants is independently and jointly associated with the entire progression spectrum of cardia lesions to cancer, particularly CO and PM_2.5_. These results reinforce the imperative to integrate air quality control into comprehensive cancer prevention strategies and highlight the need for longitudinal validation and further mechanistic research to unravel pollutant-driven pathways in gastric carcinogenesis.

## Supplementary Information

Additional file 1: Tables S1–S5. Table S1 Distribution of 5-year averaged concentrations of air pollutants among cardia lesions. Table S2 Sensitivity analysis of the relationship between long-term air pollution exposure and cardia lesion progression. Table S3 Sensitivity analysis of the association between air pollutant exposure and cardia disease progression in different exposure windows. Table S4 Sensitivity analysis of the association between air pollutant exposure and cardia disease progression after excluding different years of enrollment. Table S5 Sensitivity analysis of the association between air pollutant exposure and cardia disease progression by linear regression.

## Data Availability

The data that support the findings of this study are available from the corresponding author upon reasonable request.

## Abbreviations

AP: Attributable proportion
BMI: Body mass index
CGC: Cardia gastric cancer
CHAP: ChinaHighAirPollutants
CI: Confidence interval
HGIN: High-grade intraepithelial neoplasia
H. pylori: Helicobacter pylori
IARC: International Agency for Research on Cancer
ICD: International Classification of Diseases
LGIN: Low-grade intraepithelial neoplasia
PAHs: Polycyclic aromatic hydrocarbons
RERI: Relative excess risk due to interaction
ROS: Reactive oxygen species
SD: Standard deviation
SI: Synergy index
WQS: Weighted quantile sum
