# Supplementary material for: Long-term exposure to ambient air pollution and sequential carcinogenesis in the cardia gastric: a cross-sectional study
Source: BMC Med. 2025 Dec 22;23:708. doi: 10.1186/s12916-025-04582-1 (PMC12750796; doi:10.1186/s12916-025-04582-1)
Supplement: Supplementary file 1 — Additional file 1: Tables S1–S5. Table S1 Distribution of 5-year averaged concentrations of air pollutants among cardia lesions. Table S2 Sensitivity analysis of the relationship between long-term air pollution exposure and cardia lesion progression. Table S3 Sensitivity analysis of the association between air pollutant exposure and cardia disease progression in different exposure windows. Table S4 Sensitivity analysis of the association between air pollutant exposure and cardia disease progression after excluding different years of enrollment. Table S5 Sensitivity analysis of the association between air pollutant exposure and cardia disease progression by linear regression. [file 12916_2025_4582_MOESM1_ESM.docx]

**Additional file 1**

**Table S1.** Distribution of 5-year averaged concentrations of air pollutants among cardia lesions.

**Table S2.** Sensitivity analysis of the relationship between long-term air pollution exposure and cardia lesion progression.

**Table S3.** Sensitivity analysis of the association between air pollutant exposure and cardia disease progression in different exposure windows.

**Table S4.** Sensitivity analysis of the association between air pollutant exposure and cardia disease progression after excluding different years of enrollment.

**Table S5.** Sensitivity analysis of the association between air pollutant exposure and cardia disease progression by linear regression.

**Table S1. Distribution of 5-year averaged concentrations of air pollutants among cardia lesions.**

| **Characteristics** | | **N** | **Mean (SD)** | **Min** | **P_25_** | **Median** | **P_75_** | **Max** | **IQR** | ***P*** |
| --- | --- | --- | --- | --- | --- | --- | --- | --- | --- | --- |
| **PM_10_ (**μg/m³) | |  |  |  |  |  |  |  |  | <0.001 |
|  | Overall | 99493 | 46.207 (6.896) | 29.873 | 40.800 | 45.515 | 51.462 | 66.265 | 10.662 |  |
|  | Normal | 96216 | 46.165 (6.897) | 29.873 | 40.782 | 45.481 | 51.373 | 66.265 | 10.592 |  |
|  | Inflammation or polyp | 2881 | 47.706 (6.725) | 31.852 | 41.987 | 47.893 | 53.770 | 61.323 | 11.783 |  |
|  | pre-cancerous condition | 181 | 46.114 (6.647) | 32.868 | 40.468 | 45.173 | 51.717 | 60.582 | 11.248 |  |
|  | Pre-cancerous lesion | 78 | 45.474 (7.416) | 34.065 | 40.027 | 43.570 | 52.043 | 61.317 | 12.016 |  |
|  | Cardia gastric cancer | 137 | 45.072 (6.393) | 33.127 | 40.305 | 44.703 | 49.162 | 59.305 | 8.857 |  |
| **PM_2.5_ (**μg/m³) | |  |  |  |  |  |  |  |  | <0.001 |
|  | Overall | 99493 | 26.393 (4.141) | 16.543 | 23.195 | 26.103 | 29.723 | 37.738 | 6.528 |  |
|  | Normal | 96216 | 26.360 (4.139) | 16.543 | 23.170 | 26.087 | 29.682 | 37.738 | 6.512 |  |
|  | Inflammation or polyp | 2881 | 27.518 (4.071) | 17.504 | 23.993 | 27.597 | 31.227 | 34.920 | 7.233 |  |
|  | pre-cancerous condition | 181 | 26.269 (3.926) | 17.504 | 23.202 | 25.827 | 29.710 | 34.092 | 6.508 |  |
|  | Pre-cancerous lesion | 78 | 25.997 (4.355) | 18.405 | 22.654 | 24.598 | 29.948 | 34.543 | 7.293 |  |
|  | Cardia gastric cancer | 137 | 25.875 (3.963) | 17.391 | 23.117 | 25.642 | 28.570 | 34.400 | 5.453 |  |
| **SO_2_ (**μg/m³) | |  |  |  |  |  |  |  |  | <0.001 |
|  | Overall | 99493 | 8.322 (0.696) | 5.358 | 7.890 | 8.328 | 8.763 | 11.507 | 0.873 |  |
|  | Normal | 96216 | 8.319 (0.696) | 5.358 | 7.888 | 8.323 | 8.760 | 11.507 | 0.872 |  |
|  | Inflammation or polyp | 2881 | 8.446 (0.687) | 5.371 | 8.007 | 8.422 | 8.842 | 10.865 | 0.835 |  |
|  | pre-cancerous condition | 181 | 8.225 (0.640) | 5.817 | 7.850 | 8.275 | 8.610 | 9.788 | 0.760 |  |
|  | Pre-cancerous lesion | 78 | 8.268 (0.750) | 5.817 | 7.881 | 8.202 | 8.801 | 10.022 | 0.920 |  |
|  | Cardia gastric cancer | 137 | 8.316 (0.706) | 6.366 | 7.850 | 8.298 | 8.825 | 9.987 | 0.975 |  |
| **NO_2_ (**μg/m³) | |  |  |  |  |  |  |  |  | 0.001 |
|  | Overall | 99493 | 26.740 (6.195) | 11.295 | 21.887 | 27.172 | 32.355 | 46.530 | 10.468 |  |
|  | Normal | 96216 | 26.728 (6.203) | 11.295 | 21.861 | 27.117 | 32.365 | 46.530 | 10.504 |  |
|  | Inflammation or polyp | 2881 | 27.188 (5.908) | 12.375 | 22.530 | 27.947 | 32.243 | 43.037 | 9.713 |  |
|  | pre-cancerous condition | 181 | 26.838 (5.835) | 12.917 | 22.247 | 26.585 | 32.082 | 37.438 | 9.835 |  |
|  | Pre-cancerous lesion | 78 | 26.565 (6.301) | 15.323 | 21.774 | 24.978 | 32.294 | 38.788 | 10.520 |  |
|  | Cardia gastric cancer | 137 | 25.655 (6.232) | 12.677 | 19.993 | 25.620 | 31.140 | 36.607 | 11.147 |  |
| **O_3_ (**μg/m³) | |  |  |  |  |  |  |  |  | <0.001 |
|  | Overall | 99493 | 100.515 (6.099) | 88.037 | 96.302 | 99.438 | 104.328 | 117.658 | 8.027 |  |
|  | Normal | 96216 | 100.485 (6.070) | 88.037 | 96.302 | 99.438 | 104.233 | 117.658 | 7.932 |  |
|  | Inflammation or polyp | 2881 | 101.564 (6.912) | 88.037 | 96.348 | 99.480 | 107.973 | 116.427 | 11.625 |  |
|  | pre-cancerous condition | 181 | 100.774 (6.872) | 88.503 | 95.927 | 99.647 | 105.072 | 114.912 | 9.145 |  |
|  | Pre-cancerous lesion | 78 | 99.777 (6.967) | 88.693 | 94.653 | 98.389 | 104.296 | 113.912 | 9.643 |  |
|  | Cardia gastric cancer | 137 | 100.252 (5.173) | 88.693 | 96.338 | 99.420 | 102.998 | 114.738 | 6.660 |  |
| **CO (**mg /m³) | |  |  |  |  |  |  |  |  | <0.001 |
|  | Overall | 99493 | 0.688 (0.067) | 0.399 | 0.658 | 0.697 | 0.730 | 0.851 | 0.072 |  |
|  | Normal | 96216 | 0.687 (0.067) | 0.399 | 0.657 | 0.697 | 0.730 | 0.851 | 0.072 |  |
|  | Inflammation or polyp | 2881 | 0.708 (0.063) | 0.429 | 0.667 | 0.708 | 0.757 | 0.844 | 0.089 |  |
|  | pre-cancerous condition | 181 | 0.680 (0.071) | 0.421 | 0.646 | 0.695 | 0.729 | 0.803 | 0.083 |  |
|  | Pre-cancerous lesion | 78 | 0.694 (0.064) | 0.451 | 0.656 | 0.700 | 0.729 | 0.819 | 0.073 |  |
|  | Cardia gastric cancer | 137 | 0.680 (0.062) | 0.424 | 0.655 | 0.688 | 0.712 | 0.815 | 0.057 |  |

SD: standard deviation. IQR: interquartile range. Pre-cancerous condition: atrophic gastritis and intestinal metaplasia. Pre-cancerous lesion: low-grade intraepithelial neoplasia (LGIN) and high-grade intraepithelial neoplasia (HGIN). *P*-values were calculated using the Kruskal–Wallis test.

**Table S2. Sensitivity analysis of the relationship between long-term air pollution exposure and cardia lesion progression.**

|  |  | **Unadjusted** |  | **Adjusted** |
| --- | --- | --- | --- | --- |
|  |  | **OR (95% CI)** |  | **OR (95% CI)** |
| **PM_10_** (μg/m^3^) | |  |  |  |
|  | Q1 | Ref |  | Ref |
|  | Q2 | 1.11 (1.00, 1.24) |  | 1.12 (1.01, 1.24) |
|  | Q3 | 1.10 (0.99, 1.23) |  | 1.11 (1.00, 1.23) |
|  | Q4 | 1.66 (1.51, 1.83) |  | 1.69 (1.53, 1.87) |
| **PM_2.5_** (μg/m^3^) | |  |  |  |
|  | Q1 | Ref |  | Ref |
|  | Q2 | 1.19 (1.07, 1.33) |  | 1.19 (1.07, 1.32) |
|  | Q3 | 1.06 (0.95, 1.18) |  | 1.07 (0.96, 1.19) |
|  | Q4 | 1.85 (1.68, 2.04) |  | 1.88 (1.70, 2.07) |
| **SO_2_** (μg/m^3^) | |  |  |  |
|  | Q1 | Ref |  | Ref |
|  | Q2 | 1.19 (1.07, 1.32) |  | 1.20 (1.08, 1.34) |
|  | Q3 | 1.36 (1.23, 1.51) |  | 1.38 (1.25, 1.53) |
|  | Q4 | 1.43 (1.29, 1.58) |  | 1.44 (1.30, 1.60) |
| **NO_2_** (μg/m^3^) | |  |  |  |
|  | Q1 | Ref |  | Ref |
|  | Q2 | 1.10 (0.99, 1.22) |  | 1.10 (0.99, 1.22) |
|  | Q3 | 1.43 (1.29, 1.57) |  | 1.43 (1.30, 1.58) |
|  | Q4 | 1.11 (1.00, 1.23) |  | 1.12 (1.01, 1.24) |
| **O_3_** (μg/m^3^) | |  |  |  |
|  | Q1 | Ref |  | Ref |
|  | Q2 | 0.98 (0.88, 1.08) |  | 0.96 (0.87, 1.06) |
|  | Q3 | 0.70 (0.63, 0.78) |  | 0.69 (0.62, 0.77) |
|  | Q4 | 1.30 (1.19, 1.43) |  | 1.30 (1.18, 1.42) |
| **CO (**mg /m^3^) | |  |  |  |
|  | Q1 | Ref |  | Ref |
|  | Q2 | 1.22 (1.10, 1.36) |  | 1.22 (1.10, 1.36) |
|  | Q3 | 1.10 (0.99, 1.23) |  | 1.12 (1.00, 1.24) |
|  | Q4 | 1.77 (1.61, 1.96) |  | 1.82 (1.64, 2.00) |

Adjusted for age, sex and *H. pylori* infection.

**Table S3. Sensitivity analysis of association between air pollutant exposure and cardia disease progression in different exposure windows.**

| **Lag years** | **Air pollutant** | **OR (95%CI)** | ***P*** |
| --- | --- | --- | --- |
| 3 | PM_10_ | 1.24 (1.20-1.28) | <0.001 |
| 3 | PM_2.5_ | 1.33 (1.29-1.38) | <0.001 |
| 3 | SO_2_ | 1.06 (1.02-1.10) | 0.001 |
| 3 | NO_2_ | 1.05 (1.01-1.09) | 0.009 |
| 3 | O_3_ | 1.14 (1.10-1.18) | <0.001 |
| 3 | CO | 1.25 (1.20-1.29) | <0.001 |
| 3 | WQS | 1.11(1.08, 1.15) | <0.001 |
| 5 | PM_10_ | 1.21 (1.17-1.25) | <0.001 |
| 5 | PM_2.5_ | 1.28 (1.23-1.32) | <0.001 |
| 5 | SO_2_ | 1.17 (1.13-1.21) | <0.001 |
| 5 | NO_2_ | 1.07 (1.03-1.10) | <0.001 |
| 5 | O_3_ | 1.16 (1.12-1.20) | <0.001 |
| 5 | CO | 1.36 (1.30-1.41) | <0.001 |
| 5 | WQS | 1.10(1.07, 1.13) | <0.001 |
| 7 | PM_10_ | 1.20 (1.16-1.24) | <0.001 |
| 7 | PM_2.5_ | 1.25 (1.21-1.30) | <0.001 |
| 7 | SO_2_ | 1.30 (1.26-1.35) | <0.001 |
| 7 | NO_2_ | 1.08 (1.05-1.12) | <0.001 |
| 7 | O_3_ | 1.14 (1.10-1.18) | <0.001 |
| 7 | CO | 1.44 (1.37-1.51) | <0.001 |
| 7 | WQS | 1.11(1.08, 1.15) | <0.001 |
| 10 | PM_10_ | 1.19 (1.15-1.23) | <0.001 |
| 10 | PM_2.5_ | 1.22 (1.18-1.27) | <0.001 |
| 10 | SO_2_ | 1.24 (1.20-1.29) | <0.001 |
| 10 | NO_2_ | 1.07 (1.03-1.11) | <0.001 |
| 10 | O_3_ | 1.10 (1.07-1.14) | <0.001 |
| 10 | CO | 1.47 (1.40-1.55) | <0.001 |
| 10 | WQS | 1.10(1.07, 1.14) | <0.001 |

**Table S4. Sensitivity analysis of the association between air pollutant exposure and cardia disease progression after excluding different years of enrollment.**

| **Excluded years** | **Air pollutant** | **OR (95%CI)** | ***P*** |
| --- | --- | --- | --- |
| All participants | PM10 | 1.21 (1.17-1.25) | <0.001 |
| All participants | PM2.5 | 1.28 (1.23-1.32) | <0.001 |
| All participants | SO2 | 1.17 (1.13-1.21) | <0.001 |
| All participants | NO2 | 1.07 (1.03-1.10) | <0.001 |
| All participants | O3 | 1.16 (1.12-1.20) | <0.001 |
| All participants | CO | 1.36 (1.30-1.41) | <0.001 |
| All participants | WQS | 1.10 (1.07-1.13) | <0.001 |
| Excluded participants enrolled in 2022 | PM10 | 1.14 (1.08-1.19) | <0.001 |
| Excluded participants enrolled in 2022 | PM2.5 | 1.21 (1.15-1.27) | <0.001 |
| Excluded participants enrolled in 2022 | SO2 | 1.16 (1.10-1.22) | <0.001 |
| Excluded participants enrolled in 2022 | NO2 | 0.97 (0.93-1.02) | 0.250 |
| Excluded participants enrolled in 2022 | O3 | 1.11 (1.06-1.17) | <0.001 |
| Excluded participants enrolled in 2022 | CO | 1.27 (1.20-1.34) | <0.001 |
| Excluded participants enrolled in 2022 | WQS | 1.06 (1.02-1.11) | 0.007 |
| Excluded participants enrolled in 2023 | PM10 | 1.30 (1.23-1.37) | <0.001 |
| Excluded participants enrolled in 2023 | PM2.5 | 1.39 (1.31-1.46) | <0.001 |
| Excluded participants enrolled in 2023 | SO2 | 1.20 (1.14-1.26) | <0.001 |
| Excluded participants enrolled in 2023 | NO2 | 1.17 (1.11-1.23) | <0.001 |
| Excluded participants enrolled in 2023 | O3 | 1.11 (1.06-1.17) | <0.001 |
| Excluded participants enrolled in 2023 | CO | 1.27 (1.20-1.34) | <0.001 |
| Excluded participants enrolled in 2023 | WQS | 1.13 (1.09-1.18) | <0.001 |

**Table S5. Sensitivity analysis of the association between air pollutant exposure and cardia disease progression by linear regression.**

| **Sensitivity analysis** | **β** | **95%CI** | ***P*** |
| --- | --- | --- | --- |
| **Assuming** **equal spacing among stages** | | | |
| **PM_10_** | 0.0008 | (0.0006, 0.0010) | < 0.001 |
| **PM_2.5_** | 0.0017 | (0.0014, 0.0021) | < 0.001 |
| **SO_2_** | 0.0066 | (0.0044, 0.0089) | < 0.001 |
| **NO_2_** | 0.0002 | (0.0000, 0.0005) | 0.071 |
| **O_3_** | 0.0008 | (0.0005, 0.0010) | < 0.001 |
| **CO** | 0.1212 | (0.0978, 0.1445) | < 0.001 |
| **Assuming** **proportional spacing among stages (1.5)** | | | |
| **PM_10_** | 0.0003 | (0.0002, 0.0005) | < 0.001 |
| **PM_2.5_** | 0.0008 | (0.0005, 0.0011) | < 0.001 |
| **SO_2_** | 0.0032 | (0.0015, 0.0049) | < 0.001 |
| **NO_2_** | 0.0001 | (-0.0001, 0.0002) | 0.595 |
| **O_3_** | 0.0004 | (0.0002, 0.0005) | < 0.001 |
| **CO** | 0.0579 | (0.0402, 0.0756) | < 0.001 |
